# Advancing the development of new tuberculosis treatment regimens: The essential role of translational and clinical pharmacology and microbiology

**DOI:** 10.1371/journal.pmed.1002842

**Published:** 2019-07-05

**Authors:** Kelly E. Dooley, Debra Hanna, Vidya Mave, Kathleen Eisenach, Radojka M. Savic

**Affiliations:** 1 Department of Medicine, Johns Hopkins University School of Medicine, Baltimore, Maryland, United States of America; 2 Bill & Melinda Gates Foundation, Seattle, Washington, United States of America; 3 Byramjee Jeejeebhoy Government Medical College–Johns Hopkins University Clinical Research Site, Pune, India; 4 TB or NOT TB Consulting, LLC, Little Rock, Arkansas, United States of America; 5 University of California San Francisco, San Francisco, California, United States of America

**Summary points**

- Translational and clinical pharmacology are the state-of-the-art tools used by drug developers to efficiently move compounds and regimens through all drug development phases. Tuberculosis drug and regimen development, though, has traditionally underutilized these modern, model-based drug development approaches, despite the urgent need to understand major pharmacological aspects not only of the new candidates but also of existing drugs.
- Translational platforms that include drug combinations are critical and should encompass data from multiple preclinical drug development tools (in vitro and in vivo models) to select the best regimens to be moved forward into clinical development.
- Quantitative pharmacokinetic (PK)–pharmacodynamic (PD) approaches should be incorporated into all phases of drug development and be used for selection of optimal dose and schedule, assessment of drug–drug interactions, and dose determination in key populations including pregnant women, children, and people living with HIV. Quantitative pharmacology models should further be utilized for clinical trial design using clinical trial simulations.
- Microbiology determinants such as precisely assessed minimum inhibitory concentrations (MICs) as well as quantitative longitudinal cultures integrated with PK-PD assessment will substantially inform and enhance all phases of drug development.
- Commitment of all stakeholders, data sharing, and resource investment are required for development and utilization of these tools, which are necessary for successful TB regimen development.

## Introduction

**Fig 1:**
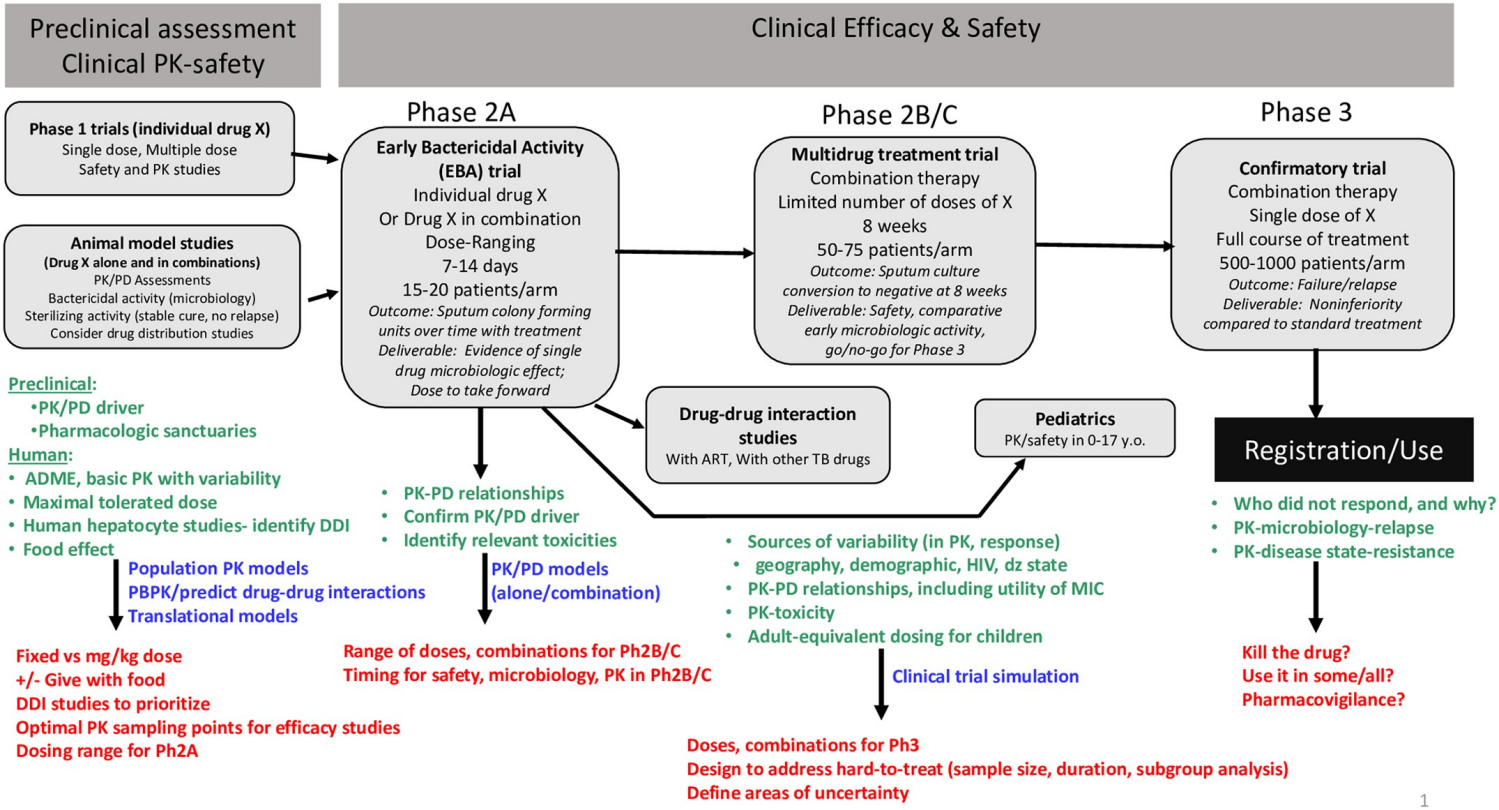
Schema of preclinical and clinical pharmacology studies important for TB drug and regimen development. By phase of development, in green are the questions to be addressed, in blue are the tools to use to answer the questions, and in red are the outputs. ADME, absorption, distribution, metabolism, excretion; DDI, drug–drug interaction; Dz, disease; MIC, minimum inhibitory concentration; PBPK, physiologically based PK; PD, pharmacodynamic; Ph2A, phase 2A; Ph2B/C, phase 2B and C; Ph3, phase 3; PK, pharmacokinetic; TB, tuberculosis; y.o., year-olds.

Application of clinical pharmacology best practices is essential to the efficient and rational development of drugs. In general, knowledge gained about exposure–response relationships in preclinical models aids drug and dose selection in human studies, and biomarkers and pharmacokinetic (PK) data one collects in early to middle drug development can be used to predict the dose and treatment response of promising therapeutics in definitive phase 3 trials. The essentiality of sound clinical pharmacology in tuberculosis (TB) drug and regimen development is heightened by unique challenges in assessing drugs for this disease—aspects of the organism’s biology, the variability in lung pathology, uncertainties about how to link treatment outcomes seen in preclinical models with those seen in humans (which thwarts preclinical–clinical translational work), the lack of predictive early clinical biomarkers, and the high variability in treatment response across patients and populations (Fig 1). In TB disease, *Mycobacterium tuberculosis* (*M*.*tb*) bacilli are detected in necrotic granulomas, large cavities with liquefied contents, and intracellularly within macrophages. We believe that drugs must access each of these compartments to achieve cure in patients [1]. We also believe that TB drugs and regimens must kill bacilli in different metabolic states, from actively multiplying to semidormant [2,3]. Both in vitro and in vivo preclinical models are leveraged to assess the clinical utility of new TB drugs and drug combinations. These models vary both in their ability to assess efficacy relative to the shifting metabolic states of *M*.*tb* infection and in their ability to recapitulate human disease. Still, two models are proving to be highly informative. The mouse model of infection has been invaluable in selecting rank-ordered drug combinations, whereas the now-validated in vitro pharmacodynamic (PD) system (IVPDS, or “hollow fiber model” for TB) has significantly improved our understanding of the PK drivers of treatment response in various growth and physiologic states [4,5]. In the IVPDS, an elaborate system of dialysis-like tubing allows the investigator to reproduce human-like concentration–time curves and see how different PK profiles affect killing of bacilli that are living in the system. Dose-fractionation studies, for example, can be carried out, and one can determine whether a drug’s activity is time dependent or, rather, concentration dependent. Or one can test a drug’s activity when the organism is nutrient starved, in log-phase growth, or intracellular. Whereas these models are informative, there remain gaps in our ability to bridge preclinical and clinical data using modern translational quantitative modeling [6]. There are also gaps in our ability to link surrogate end points in early-phase clinical trials (namely, longitudinally collected sputum cultures) and clinically relevant end points of treatment failure, relapse, and death in later-phase trials [7,8]. The identification of accurate tools that identify those patients who are unlikely to achieve cure with shortened regimens (specifically, patients with a disease phenotype that is “hard to treat”) would have immense value to both clinical trialists and TB clinicians [9]. The TB clinical pharmacology field has the opportunity to apply state-of-the-art quantitative pharmacology tools to bridge preclinical and clinical data more effectively and to enhance learning across the continuum of clinical development [9–13]. In this paper, based on discussions occurring at a WHO workshop held in March 2018, we describe our views on best practices for incorporating translational, PK-PD, and microbiologic assessments into drug development [14].

## The importance of understanding PK-PD relationships by phase of regimen development

Key uncertainties and questions regarding the use of clinical and translational pharmacology, biomarkers, and microbiology in the evaluation of novel TB treatments are listed in Table 1. Herein, we review these, focusing in on the implications relevant to each developmental phase.

**Table 1:** Key uncertainties and questions about the use of clinical and translational pharmacology, biomarkers, and microbiology to advance TB treatments that were addressed at the WHO-sponsored workshop, advances in clinical trial design for development of new TB treatments. (Adapted from [15]).

Topic Area	Question
Clinical Pharmacology	What is the importance of understanding PK-PD relationships by phase of regimen development?
Pharmacometrics	How does quantitative modeling and simulation integrate PK and microbiology-based PD measures (e.g., MIC, bacterial burden as predictive covariates of treatment response) to inform drug development decision-making, especially in later stages of regimen evaluation?
Preclinical/Translational Pharmacology	Can dynamic experiment-level in vitro assessments (i.e., HFS-TB) be integrated with patient-level bacteriological data to improve quantitative clinical PK-PD predictions and streamline model development?
Biomarkers	What would be the most efficient framework for bacteriologically based biomarker identification and characterization in clinical trials to enable integration in modeling and simulation-based analyses?
Bacteriology	Should quantitative PK-PD models describing relevant bacteriologically based covariates be used to guide dose finding and dose optimization in key populations during early development?
Drug Development	How do we make use of PK-PD across clinical development phases to identify pharmacology-guided drug regimens?

Abbreviations: HFS-TB, hollow-fiber in vitro pharmacodynamic system for assessing TB drugs; MIC, minimum inhibitory concentration; PD, pharmacodynamic; PK, pharmacokinetic; TB, tuberculosis

### Preclinical drug development

Investigation of PK-PD properties of a candidate drug in the preclinical space is critical for advancing new TB drugs and for building effective combination regimens with a clear rationale for contribution of each new agent. The combination of multiple new chemical entities requires an enhanced understanding of PK-PD across the development paradigm and refined understanding of penetration and mechanism of action within the granuloma. Several experimental tools are utilized at the preclinical stage for new TB drug assessment, each providing unique results that can be used synergistically for decision-making [16]. The European Medicines Agency (EMA)-qualified in vitro hollow-fiber system generates PK-PD data that can be used to refine in vivo animal experiments [16,17]. These in vitro data are integrated with data from multiple in vivo models, which include acute, chronic, and relapsing BalbC (older, well-validated standard mouse model) and Kramnik (newer model with more human-like pathology) mouse models of infection as well as marmoset and rabbit models of disease [18,19]. All of these models provide valuable information on the drug’s potential for microbiologic activity and sterilization (killing of semidormant bacilli) but generate only limited PK-PD data [4,5]. Collectively, these data contribute to understanding spatial distribution of candidate drugs at the site of action in the lung, PK-PD relationships for a single drug, contribution of an individual drug to the entire regimen, synergies of drugs within combinations, and potential for shortening treatment duration. Systems pharmacology models or PK-PD–driven translational platforms are essential in order to adequately assess the true potential of investigational regimens. In the absence of PK-PD–driven translational data, regimens move into late-phase trials with important uncertainties (about the real likelihood that a regimen will produce cure at rates equal to or better than that of the standard-of-care regimen) that traditional microbiologic surrogate markers like sputum culture conversion cannot adequately address [6]. Further, current lack of informative, translational biomarkers that are portable across TB clinical drug development stages for regimen and dose optimization puts further weight on preclinical analyses for de-risking regimen development [20].

The best way to identify new quantitative and translational tools for discovery and optimization of new TB treatment regimens is by investing in and enhancing data collaboration and translational modeling activities. This would support development of a universal preclinical–clinical mechanistic PK-PD system for TB drug combinations with high translational and predictive features to answer questions such as the following: What is the human equivalent dose/schedule of a candidate drug used in a combination regimen that will maximize its contribution to reducing treatment duration? What is the likelihood of achieving treatment durations of 1–3 months with a putative treatment-shortening regimen? And is it possible to shorten treatment duration in all disease phenotypes and all patients? A translational, data-driven mechanistic tool would be able to predict comparative efficacy and intended treatment-shortening potential of new candidate regimens based on preclinical data and optimized translational simulations. The major features of such a translational tool would ideally include: (1) quantification of bacterial growth dynamics in the absence of treatment, (2) quantification of the immune system response in the absence and presence of treatment and as a function of bacterial load and infection time, (3) quantification of the contribution of each drug (concentration–response relationship) to the observed total efficacy of drug combinations, (4) quantification of the interplay between disease pathology and drug response including description of tissue penetration, (5) a fully estimated set of model parameters with variability and uncertainty, and (6) appropriate scaling functions to human PK and PD to allow for accurate translational simulations. Because these components span numerous approaches to drug evaluation, from in vitro studies to clinical trials, data often need to be obtained from multiple sources; once assembled through collaborations, they can permit accurate translational simulations to help address the critical questions and enable decision-making by stage–gate regimen developers (developers that have divided development into stages with go/no-go decisions at the end of each stage). Integration of these principles will allow for rational selection of the best regimens to be moved forward into clinical development, selection of rational dose ranges to be studied in clinical phases, quantitative predictions of clinical trial outcomes, and informed choice of clinical trial designs.

### Clinical PK and PD

Prior to initiating a phase 3 registration trial of a new regimen, it is also important to understand the PK of experimental drug(s), exposure–response relationships, PK–toxicity relationships, risk and magnitude of drug interactions, drug safety, sources of variability (in PK, safety, treatment response) in the population, and PK in key populations (Fig 1). Phase I trials provide basic PK and safety information. It is important from a practical standpoint to assess food effect early, as this may impact administration requirements and may complicate approaches to coadministration with companion drugs, as some are taken on an empty stomach (e.g., rifampicin), whereas others are absorbed better with food (e.g., rifapentine, delamanid, bedaquiline) [21–24]. It is also necessary to determine whether weight-based dosing will be required. The requirement for weight banding adds complexities to field implementation as well as reduces the opportunity to coformulate companion drugs into fixed-dose combinations. Additionally, caution is noted, as systematic underdosing of lower-weight individuals can occur when weight banding is used without a reliable clinical PK-PD evidence base [25].

Over the course of phase 1 and 2 testing, assessment of drug PK in geographically and ethnically diverse populations is also invaluable, as variability of drug exposures across populations has been noted [22]. Sparse PK sampling can be employed after identifying optimal sampling times, and population PK modeling is then used to identify factors associated with variability in drug exposures (e.g., sex, race, HIV coinfection, malnutrition). For example, in individuals of black race, bedaquiline exposures are 50% lower than in persons of other racial backgrounds; rifampicin concentrations are very low in children who are malnourished or who have HIV infection; and isoniazid clearance is dependent on N-acetyltransferase 2 acetylator status [22,26,27]. Drug–drug interaction studies should be pursued in middle drug development and not left for late phases of development, particularly for interactions between TB and HIV drugs. The need for drug–drug interaction studies can be assessed based on knowledge of the putative TB drug(s) and standard-of-care HIV drugs’ metabolic pathways and their proclivity for inducing or inhibiting metabolizing enzymes or transporters. If interaction studies of HIV and TB drugs are not conducted early, the impact of the new TB regimens on antiretroviral therapies (and vice versa) and the resultant effects on viral load suppression and on achieving durable cure from TB will not be understood; as a consequence, the inclusion of HIV patients into late-phase trials will be hindered, limiting the assessment of the safety, tolerability, and efficacy of the regimen in this key population. Moreover, drug–drug interaction studies will still be needed, and substudies will need to be designed, adding significant complexity and delays when they are embedded into late-phase, confirmatory clinical trials [28,29].

In early phase 2A trials, in which a drug or drug combination is administered for 7–14 days to small cohorts of patients (*n* = 15–20), a range of doses and schedules is tested for early bactericidal activity (EBA). Data on safety, tolerability, and longitudinal quantitative sputum bacillary loads are collected, and semi-intensive PK sampling is performed to characterize individual drug exposures and PK-PD relationships, which can narrow the doses to be tested in subsequent trials. In phase 2B trials, microbiologic responses to treatment are assessed through serial sputum cultures up to 8–16 weeks of treatment. In phase 2C trials, the experimental regimens are administered for their intended duration (e.g., 3 or 4 months), and patients are followed to collect information on longer-term clinical outcomes (failure, relapse, death) [30]. Such phase 2B and phase 2C studies are typically multinational and can produce rich PK and microbiologic data from geographically diverse settings. We propose that sparse PK sampling be embedded in all phase 2B/C trials. If feasible, sparse PK sampling obtained on more than one occasion—for example, early in treatment (intensive phase) and then later in treatment (continuation phase)—would allow the quantification of longitudinal drug exposures that, in turn, characterize exposure–response relationships necessary for selecting the accurate dose(s) to evaluate in phase 3 trials [13,31]. Additionally, population PK-PD modeling can define the relative contributions of factors that lead to delayed culture conversion, assessing within the model the full suite of potential features, from low drug exposures to clinical factors such as disease severity or patient characteristics. Patient and disease severity characteristics are important to incorporate into models, as the hardest-to-treat phenotypes of disease disproportionately drive the unfavorable outcomes in contemporary phase 3 trials [10,11,32]. To date, clinical PK-PD analyses have been unable to adequately inform decision-making on selecting an optimal regimen duration. A PK-PD tool that predicts the optimal treatment duration based on data from preclinical studies, phase 2 trials, and both successful and unsuccessful phase 3 trials would be extremely valuable.

In middle development (at the phase 2 stage), PK–toxicity studies are also needed to define the therapeutic margin and ensure that dose(s) used in phase 3 are likely to be safe and well tolerated. PK–safety relationships influence both dose and schedule (duration, dosing frequency), with some drugs displaying toxicity associated with cumulative exposure (e.g., linezolid, ethambutol) and others causing more adverse effects when given on an intermittent schedule (e.g., rifamycins administered thrice or once weekly) [33–35]. Overlapping toxicities can also be explored with PK data in hand to help discern relationships. For example, prolongation of the QT segment on the electrocardiogram, a cardiac toxicity that can lead to torsades de pointes, can be related to concentrations of the parent drug or metabolite and is of increased concern when QT-prolonging drugs are administered concurrently [36].

Phase 3 trials provide the first opportunity to assess drug efficacy by comprehensively collecting data on drug exposures, adherence, microbiological response over time, safety, and long-term clinical outcomes; furthermore, this often is the only setting in which reduced treatment durations are tested. Phase 3 studies also offer larger numbers of patients from key populations (e.g., people living with HIV and children). Because of these features, we recommend that phase 3 trials include sparse PK sampling whenever feasible and that samples be collected on all patients. PK-PD assessments can be performed on a subset of study participants to identify the reasons for poor treatment outcomes. If the trial was successful, these samples would allow analyses that inform future use and scale-up of the regimens; if the trial was not successful, these data help ascertain the reason(s) why and are critical for determining next steps.

## Microbiology and quantitative pharmacology

Although microbiology (e.g., in vitro minimum inhibitory concentration [MIC]) is widely accepted as an important determinant of response to treatment, integrated PK and microbiology-based PD measures built into late-stage clinical trials to confirm relationships are rarely undertaken. MICs are assessed in preclinical drug development, and the choice of dose and schedule is driven by the desire, for example, to maintain plasma drug levels above MIC for a defined duration. This approach has shortcomings, as PK-PD indices are often derived based on plasma PK, which is often suboptimal compared with the site-of-action PK; traditional assessment of MIC usually lacks precision [37], and by definition, MIC values indicate inhibition of bacterial growth rather than bacterial killing, which is key for cure. Bacteria with higher MIC are harder to eradicate, and patients with high-MIC bacteria might need more aggressive regimens or longer treatment to achieve cure. Similarly, pretreatment bacterial burden in sputum is highly associated with treatment response. The recent TB Reanalysis of Fluoroquinolone Clinical Trials (TB ReFLECT) meta-analysis revealed that patients with low bacterial burden at baseline could be effectively treated with a shortened (4 month)-duration experimental regimen [9]. Similarly, time-to-culture conversion on standard treatment appears to be shorter in patients with low baseline bacillary load. However, a number of questions remain unanswered—Is there a correlation between baseline bacterial burden and MIC? Does MIC change over time with treatment? Under which circumstances can higher bacterial load or MIC be overcome with higher doses, strong companion drugs, or longer treatment? Should we index PK parameters (Cmax, area under the concentration–time curve [AUC]) to MIC or to a data-informed factor of the MIC?

To address these questions, collection of microbiology data is key in all stages of clinical trials, especially late-stage trials followed up with adequate analysis. Collecting sputum specimens for MIC and bacterial load determinations in clinical drug development so that their value (in subsequent studies and in clinical practice) can be determined is important. *M*.*tb* isolates should be available for MIC determination from baseline and last positive cultures, and sputum specimens should be assessed over time for changes in bacterial load. Knowledge of strain lineage (e.g., Haarlem, Latin American/Mediterranean, W/Beijing) may also be helpful, as there may be strain heterogeneity with regard to virulence and drug response. A standardized method for providing robust and accurate MIC determinations, such as the 14-drug microtiter plate (ThermoFisher) employed by the Comprehensive Resistance Prediction for Tuberculosis: An International Consortium (CRyPTIC) [38], should be used. Techniques for measuring bacterial burden including time to positivity in mycobacterial growth indicator tube (MGIT) culture, cycle threshold in GeneXpert MTB/RIF assay, or potential novel biomarkers (e.g., quantification of sputum lipoarabinomannan [LAM] levels) should be routinely included in clinical trials to enable investigation of predictive bacterial burden biomarkers [39,40]. Assays such as GeneXpert cycle threshold have the advantage of producing results in real time, though one disadvantage is that DNA from both live and dead bacilli can be detected. Lastly, it is important to align new drugs with new diagnostics. Specifically, detection and characterization of resistance is a key component of TB drug development, and whole-genome sequencing can identify mutations that are associated with decreased susceptibility of *M*.*tb* strains to new drugs.

## Key populations: Optimal design to extend treatment advances to all

Young children and pregnant women with TB may be at particularly high risk of adverse outcomes resulting from inadequate TB treatment [41,42]. There are limited data to inform use of TB drugs in pregnant women because they are routinely excluded from clinical trials, and there is no requirement to study them from any regulatory authority. This may change with the report of the Task Force on Research Specific to Pregnant Women (PRGLAC) released in September 2018 [43]. In Europe, a pediatric investigation plan (PIP) is required for drug registration, but there is no requirement for data from the pediatric patient population. In the United States, because TB is considered an orphan disease, the Pediatric Research Equity Act (PREA) does not apply to drugs developed for TB, relieving companies of the requirement to study TB drugs in children for registration. As it may be difficult to recruit children and pregnant women with TB in any given location, design of clinical trials in these populations must be optimized for efficiency and yield of safety and PK data that will be needed to support dosing recommendations. Full efficacy trials are generally not required [44].

### Children

Opening doses in different pediatric age cohorts are more likely to be accurate when based on models that incorporate adult PK data and information about developmental pharmacology and when evidence-based target PK ranges are defined explicitly, rather than relying on empiric dose selection (e.g., same mg/kg dose as adults) [45]. Given that drug disposition is most variable between the ages of 0 and 2 years (and changes especially rapidly in the first 3–6 months of life), we suggest including a larger number of children in the youngest cohort to ensure full knowledge of drug disposition in that rapidly developing age group; the sample size of adolescents can be relatively smaller because drug disposition is similar in teens and adults. Key features of a pediatric PK–safety study include model-informed initial dose selection; early interim analysis of PK results in each age cohort (with dose adjustment and model updating); use of optimal sampling theory, a data-driven approach that informs the selection of the most informative time points for PK sampling while minimizing the number of required samples; defining the timing and content of safety visits to reflect knowledge of each drug’s preclinical toxicology and adult toxicity information; clear and evidence-based selection of PK target ranges for parent drug and metabolite(s); and model-based analysis of data by a pharmacometrician.

### Pregnant women

Studies in pregnant women should take into account pregnancy-related physiologic changes, including changes in renal clearance, drug metabolism, and protein binding [46,47]. Some metabolizing enzymes have higher activity during pregnancy (cytochrome [CYP] P450 2A6, 3A4, 2D6; uridine 5'-diphospho-glucuronosyltransferase [UGT] 1A4), whereas others have lower activity (CYP1A2, 2C19); the magnitude of difference in enzyme activities in pregnant versus nonpregnant women differs by trimester [48]. As selection of doses most likely to achieve (but not exceed or fall significantly short of) therapeutic targets is especially crucial in pregnant women with TB, model-based dose selection is best from both scientific and ethical standpoints. PK assessments should be performed in the second and third trimester and then postpartum so that timing of dose adjustments can be assessed. Depending on the duration for which a given drug is administered, each woman may serve as her own control, reducing variability. Pharmacometric modeling should be used in the analysis so that specific effects of pregnancy on drug absorption, distribution, and clearance can be estimated while considering other cofactors that may affect the drug’s disposition, and recommendations for dose adjustments can be made with maximal knowledge. With regard to safety, whereas a very strong safety signal may be detected in a study powered to detect PK changes, a much larger cohort of women is needed to characterize the full safety profile of a drug in pregnancy for the mother and fetus. Pharmacovigilance via pregnancy registries is one way to achieve this (e.g., http://www.apregistry.com/ for antiretrovirals).

## Site-of-disease PK: Relevance for drug development and optimization

*M*.*tb* bacilli are present in multiple compartments in a patient with pulmonary TB but are most numerous in large cavitary lesions that contain liquefied, caseous material. To effect cure, it is currently believed that drugs must penetrate necrotic granulomas and cavitary lesions to inhibit or kill the viable bacilli that are not expelled by coughing. We should ensure that the drugs achieve adequate bactericidal concentrations in the lesions where bacilli are present. Preclinical and clinical research focused on drug quantification in these matrices may help inform regimen selection for treatment-shortening trials, including drugs, doses, duration, and companion drugs. This is an area in which translational PK-PD research may be particularly valuable. In rabbit models of pulmonary TB that have human-like pathology, it has been observed that some drugs have excellent penetration into lesions, as assessed spatially by matrix assisted laser desorption/ionization (MALDI) mass spectrometry or quantitatively by laser capture dissection and laser capture microdissection liquid chromatography mass spectrometry (LCM-LC/MS), whereas others display poor lesion penetration [49–51]. Of note, all four current first-line TB drugs reach therapeutic concentrations in TB lung lesions [52]. Patients with highly drug resistant TB who must undergo lung resection for cure have participated in research aimed at measuring drug concentrations in lung compartments following observed dosing [1]; data from such investigations help bridge preclinical and clinical studies and provide evidence concerning the contribution of drug PK to acquisition of drug resistance in lung microenvironments [53]. With rabbit lesion penetration data for a novel drug as well as data on human plasma PK and treatment outcomes, translational models can be built that shed light on the differential response to TB treatment that results from differences in lung pathology [19]. These strategies may help reduce the risk of late-phase failure for drugs with promising preclinical and early clinical microbiologic efficacy by identifying early those compounds with poor penetration into critical lung lesions. Translational models may also be important in developing therapies for other manifestations of TB disease, like central nervous system or extrapulmonary TB.

## Conclusions

Modern drug development tools using quantitative and translational pharmacology and microbiology are proving to be invaluable when applied to TB drug and regimen development programs. We can further improve on these tools by constructing predictive, fully translational models that fully integrate data and knowledge from diverse models and sources including in vitro susceptibility data, drug(s) mechanism-of-action characteristics, hollow-fiber model PK-PD data, cure results from multidrug studies in different animal models, phase 2 longitudinal microbiologic data, and information on PK-PD, adherence, and well-defined clinical outcomes from carefully conducted phase 3 trials (Table 2). Comorbidities, sites of disease, characteristics of the infecting strain, and host immune status are also highly relevant; information on these elements can further enhance model performance. For the clinical phases of development, studies of drug interactions with relevant ART agents should be conducted early to allow the inclusion of HIV-infected patients in definitive trials. Similarly, children and pregnant women with TB should also be included in well-designed safety and PK studies.

**Table 2:** Use of PK-PD, microbiology, and biomarkers in TB regimen development: Required elements, recommended but optional components, and research gaps (adapted from World Health Organization [15]).

Question	Consensus	Options	Research
**What is the importance of understanding PK-PD relationships by phase of regimen development?**	PK studies should be included throughout drug/regimen development phases, in both early and late stages of development. PK samples should be collected in all treatment trials with clear documentation of dosing history.	Other PK studies should be performed in the spirit of modern drug development, including the following:	Optimal timing and frequency of PK sampling by type of trial (e.g., phase 2A, 2B, 2C) to yield the most information in the most efficient way.
	A guidance that outlines information to be collected and parameters to be identified at each phase of drug development is needed. This guidance should be organized by sections of minimum information and optimal information. This could be undertaken by a group of individuals with expertise in PK-PD research, such as the WHO Task Force on the PK-PD of TB medicines.	Drug–drug interaction studies, especially with companion TB drugs or antiretrovirals.	Translational modeling and quantitative pharmacology to link preclinical, early-mid clinical (with microbiology outcomes). and definitive trial (with clinical outcomes) results. Role of clinical trial simulation with phase 2 data to inform phase 3 design.
	Importance of PK in phase 2 trials to allow understanding of dose–exposure–response relationships for dose selection in definitive trials.	Evaluation of PK–toxicity relationships for key toxicity concerns (e.g., QTc).	Validation and refinement of translational tools and modeling activities (mouse model, HFS, systems pharmacology model) through data sharing.
	Critical importance of PK–safety assessment in phase 2/3 to inform the need for dose/schedule adjustments. Particularly important for narrow therapeutic index drugs.	Sparse PK collection in phase 3 to strengthen population PK modeling and to explore exposure differences in relevant subgroups including poor responders.	Biomarker (host, microbiology) explorations to find better ways to identify best regimens to carry forward from middle drug development.
	Population PK modeling to understand sources of variability (e.g., sex, race, age, HIV status) in drug exposures and response.		
	Phase 2B/C studies with arms testing different doses and duration and collection of treatment outcomes will be most informative for identifying regimens most likely to be successful for treatment shortening.		
**How does quantitative modeling and simulation integrate PK and microbiology-based PD measures (e.g., MIC, bacterial burden as predictive covariates of treatment response) to inform drug development decision-making, especially in later stages of regimen evaluation?**	Importance of gaining a better understanding of the relevance and value of MIC measurements as well as baseline quantitative bacterial burden in assessments of exposure–response relationships.	*M*. *tuberculosis* isolates should be stored, including at a minimum the baseline isolate and that of the last positive culture.	Key research questions to answer by quantitative pharmacology by time of registration:
	Collection of specimens for MIC (genotypic, phenotypic, whole-genome sequencing, etc.) in clinical drug development will allow for value assessment. Isolates should be collected at baseline and during midterm and late-stage development.	Bacterial burden should be quantified longitudinally via collection of serial sputum samples.	PK-PD underpinnings to support dose recommendations, including in hard-to-treat patients and key populations.
	Specific guidance from WHO PK-PD Task Force to provide details on standardized approaches for collection of isolates (which isolates, how to collect, how to store, when to collect, what type of assay would be needed)		PK–toxicity relationships.
			Drug–drug interactions with companion TB and HIV drugs.
			Evaluation of value of MIC (static drug concentration in relevant medium) versus dynamic susceptibility information in drug and regimen assessment.
**Can dynamic experiment-level in vitro assessments (e.g., HFS) be integrated with patient-level microbiology data to improve quantitative clinical PK-PD predictions and streamline model development?**		Investment in development of translational tools and modeling activities (mouse model, HFS, systems pharmacology model) that can inform regimen composition.	
**What would be the most efficient framework for microbiology-based biomarker identification and characterization in clinical trials to enable integration in modeling and simulation-based analyses?**	Development and validation of novel biomarkers should be integrated in all PK-PD activities to allow for rapid assessment of the biomarkers and properties of future potential surrogates for bacterial load.		Culture-free (and sputum-free) systems as alternatives to existing culture-based systems are urgently needed.
**Should quantitative PK-PD models describing relevant microbiology-based covariates be used to guide dose finding and dose optimization in key populations during early development?**	Design of studies in key populations should be supported by clinical pharmacology principles (dosing regimen) and aided by model-based design.		

Abbreviations: HFS, hollow fiber system; MIC, minimum inhibitory concentration; PD, pharmacodynamic; PK, pharmacokinetic; QTc, corrected QT interval on electrocardiogram; TB, tuberculosis

## Abbreviations

ADME: absorption, distribution, metabolism, excretion
AUC: area under the concentration–time curve
CRyPTIC: Comprehensive Resistance Prediction for Tuberculosis: An International Consortium
CYP: cytochrome
EBA: early bactericidal activity
EMA: European Medicines Agency
HFS: hollow fiber system
IVPDS: in vitro PD system
LAM: lipoarabinomannan
LCM-LC/MS: laser capture microdissection liquid chromatography mass spectrometry
*M.tb*: *Mycobacterium tuberculosis*
MALDI: matrix assisted laser desorption/ionization
MGIT: mycobacterial growth indicator tube
MIC: minimum inhibitory concentration
PD: pharmacodynamic
PIP: pediatric investigation plan
PK: pharmacokinetic
PREA: Pediatric Research Equity Act
PRGLAC: Task Force on Research Specific to Pregnant Women
QTc: corrected QT interval on electrocardiogram
TB: tuberculosis
TB ReFLECT: TB Reanalysis of Fluoroquinolone Clinical Trials
UGT: uridine 5'-diphospho-glucuronosyltransferase
